# Retroperitoneal lymph node dissection for growing teratoma syndrome in testicular cancer: a systematic review of surgical outcomes

**DOI:** 10.1007/s00345-026-06207-5

**Published:** 2026-01-13

**Authors:** Alberto Costa Silva, Afonso Morgado, João Alturas Silva, Pedro Oliveira, Noel Clarke, Rui Almeida Pinto, Aziz Gulamhusein

**Affiliations:** 1 University Hospital Centre of São João, Alameda Professor Hernâni Monteiro, 4200-319 Porto, Portugal; 2 Faculty of Medicine, University of Porto, RISE – Health Research Network, Porto, Portugal; 3 The Christie NHS Foundation Trust, Manchester, UK

**Keywords:** Testicular cancer, Teratoma, Retroperitoneal neoplasms, Lymph node excision

## Abstract

**Purpose:**

Growing Teratoma Syndrome (GTS) is a rare entity occurring in patients with non-seminomatous germ cell tumours (NSGCT) following chemotherapy. GTS is resistant to chemotherapy and radiotherapy, making retroperitoneal lymph node dissection (RPLND) the mainstay of treatment. This review aims to synthesize evidence on the surgical management and oncological outcomes of retroperitoneal GTS.

**Methods:**

A systematic review was conducted in accordance with PRISMA guidelines and registered in PROSPERO (CRD420251233173). PubMed and Embase were searched from inception to November 2025. Risk of bias was assessed using the ROBINS-I tool.

**Results:**

Fifteen studies comprising 156 patients with NSGCT-associated retroperitoneal GTS treated with RPLND were included. The reported incidence ranged from 2.8% to 7.6%. Median patient age across studies was 16–38 years. Mixed NSGCT was present in 73–100% of orchidectomy specimens, with a teratoma component identified in 40–100%. The interval to RPLND ranged from 2 to 30 months and median operative time ranged from 160 to 432 min, and estimated blood loss from 225 to 2500 mL. Adjunctive procedures were required in 10–100% of patients, and postoperative complications occurred in 12.5–44%, with Clavien-Dindo ≥ III complications in 12.5–25%. Median length of hospital stay ranged from 5 to 15 days. Disease-free survival varied between 41.7% and 100%, and overall survival between 73.7% and 100% with follow-up ranging from 8 to 103 months.

**Conclusions:**

RPLND is a complex procedure with effective oncological outcomes for GTS. Multicentric registries are needed for generating high-quality standardised data.

**Supplementary Information:**

The online version contains supplementary material available at 10.1007/s00345-026-06207-5.

## Introduction

Growing Teratoma Syndrome (GTS) is a rare clinical entity first described by Logothetis et al. in 1982 in the context of non-seminomatous germ cell tumours (NSGCTs) [1]. It is defined by the growth of masses during or after chemotherapy, despite normalization of serum tumour markers. Histologically, GTS is confirmed by the presence of teratoma tissue in the surgical specimen. The incidence of GTS has been reported in up to 7.6% of cases, occurring predominantly in the retroperitoneum [1, 2].

GTS pathogenesis remains poorly understood, reflecting a complex interplay of cellular differentiation, selective chemoresistance, and potential molecular reprogramming during treatment [3]. Clinically, GTS presents a management challenge. It is unresponsive to both chemotherapy and radiotherapy, and delayed recognition may result in significant morbidity due to tumour bulk or compression of adjacent structures. Moreover, mature teratoma tissue carries a risk of somatic-type malignant transformation [3, 4]. Surgical removal of retroperitoneal teratoma masses via retroperitoneal lymph node dissection (RPLND) remains the standard approach for managing GTS.

The present systematic review aims to analyse the current data on retroperitoneal GTS associated with testicular cancer, focusing on patient characteristics, RPLND metrics and oncological outcomes.

## Methods

### Evidence acquisition

This systematic review was conducted in accordance with the Preferred Reporting Items for Systematic Reviews and Meta-Analyses (PRISMA) 2020 guidelines. The review protocol was registered with the International Prospective Register of Systematic Reviews (PROSPERO; registration ID: CRD420251233173).

### Search strategy

A comprehensive literature search was carried out in PubMed/MEDLINE and Embase databases from inception to November 2025. The search strategy incorporated both free-text and MeSH terms (Supplementary material 1). Only articles published in English were considered. Reference lists of relevant articles were manually screened to identify additional eligible studies. Inclusion and eligibility criteria was determined using the PICOS framework (Supplementary material 2). Studies were excluded if they: included exclusively gynaecological or non-abdominal GTS cases; single case reports; included testicular GTS cases but did not provide granular data relevant to the review objectives; conference abstracts, or papers not published in peer-reviewed journals.

### Study selection and data extraction

Two reviewers independently screened all titles and abstracts, followed by full-text evaluation (ACS and AG). Discrepancies were resolved by a third reviewer (NC). A standardised data collection form was used to extract study characteristics (author, year, country), number of patients, age, type of primary tumour, type of chemotherapy used before RPLND, interval to RPLND (the time between orchidectomy and RPLND), surgical outcomes (RPLND time, blood loss, adjunctive procedures), complication rates (stratified by Clavien-Dindo classification), and follow-up outcomes. For continuous variables, median and range values were reported.

### Risk of bias assessment

The methodological quality of included studies was appraised using Risk of Bias In Non-randomised Studies of Interventions (ROBINS-I version 2) tool [5]. Each study was independently assessed by two reviewers (ACS and AG), and disagreements were resolved by a third party (NC). The results are presented in Supplementary Material 3.

## Results

### Search results

**Fig. 1:**
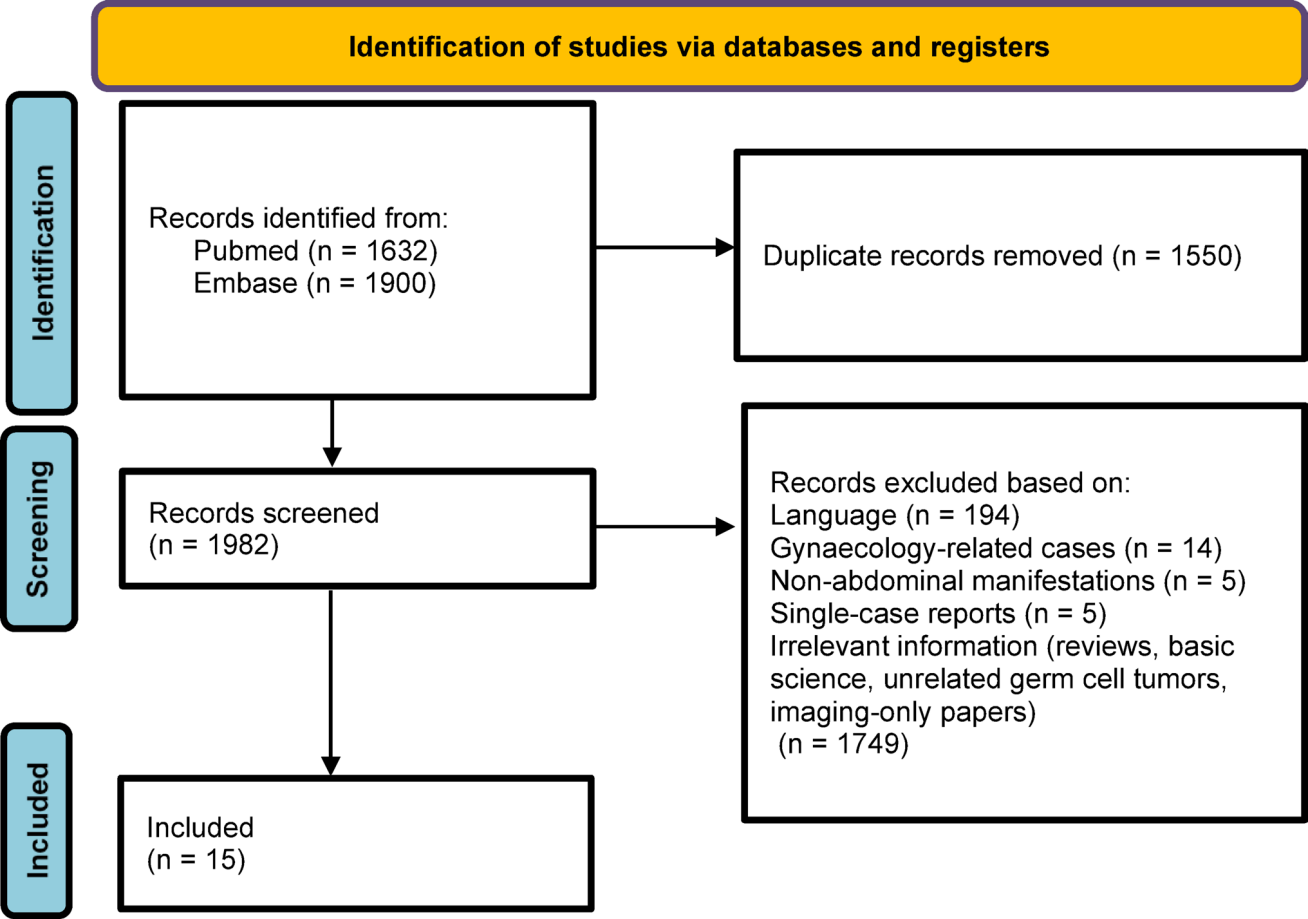
PRISMA flow diagram of study inclusion

Overall, 3532 articles were found. After screening and eligibility assessment, 15 studies met the inclusion criteria (Fig. 1), including a total of 156 patients [1, 2, 6–18]. Data spanned from 1971 to 2019, including retrospective case series. The main characteristics of the included studies are provided in Table 1.

**Table 1:** Summary of studies characteristics

Study	Country Inclusion years	Number of patients	Age years median (range)	Histology Orchidectomy, %	Interval to RPLND months median (range)	Chemotherapy	Histology RPLND (%)	Operative time minutes median (range)	EBL mL median (range)	Adjunctive procedure	Complications (CD, %)	LOS days median (range)	FU months median (range)	Outcomes
Prineethie et al., 2022 [17]	India, 2001–2019	7	27 (17–48)	Mixed NSGCT: 100%. Teratoma component: NR	NR	BEP	MT: 100%	NR	NR	NR	NR	NR	NR	All patients alive and disease-free
Hsieh et al., 2022 [18]	Taiwan, 2005–2019	2	16 (25 − 16)	Mixed NSGCT: 100% Teratoma component: NR	30 (3–56)	NR	MT: 100%	NR	NR	NR	NR	NR	77 (16–137)	All patients alive and disease-free
Acikgoz et al., 2021 [16]	Turkey, 2004–2018	12	29 (17–51)	Mixed NSGCT: 89% Teratoma component: NR MT: 11%	10 (4-104)	BEP	MT: 100%	NR	NR	NR	NR	NR	61 (8-184)	Disease-free:41.7% Relapse: 58.3% (salvage TIP) 5-year OS: 73.7%
Paffenholz et al., 2018 [14]	Germany,2000–2016	22	28 (22–34)	Mixed NSGCT: 73% Teratoma component: 40% MT: 9.1% Pure EC: 9.1% Pure YST: 9.1%	NR	BEP, EP, PEI	MT: 100%	160 (142–320)	300 (175–450)	18% -Aorta resection + graft: 2 -Cava resection + graft: 1 -Resection of renal vein + Graft:1 -Nephrectomy: 1 -Bowel resection: 1	23% CD ≥ III: 18% -Hematoma with drainage: 1 -Necrotizing pancreatitis: 1 -Bowel perforation: 1 -Bowel ischemia:1	10 (9–14)	25	Relapse: 9% Alive: 95.5%.
Scavuzzo et al., 2014 [15]	Mexico, 2014	2	26 (23–28)	Mixed NSGCT: 100% Teratoma component: 100%	9 (5–12)	BEP	MT: 100%	255 (250–360)	2500	NR	NR	5 (3–6)	9 (8–10)	All patients alive and disease-free
Lee et al., 2014 [13]	USA, 2005–2012	15	23 (16–39)	Mixed NSGCT: 80% Teratoma component:40% SGCT: 13% Unknown: 7% (after chemotherapy)	3 (2–45)	BEP	MT: 100%	372 (252–912)	600 (100–7000)	33% -Nephrectomy: 1 -Renal artery anastomosis: 1 -Renal artery embolectomy: 1 -IVC thrombectomy: 1 -Resection of iliac vein: 1	27% CD ≥ III: 13% -Arytenoid dislocation: 1 -Bilateral renal ischemia: 1	5 (3–19)	8 (5–64)	All alive and disease-free
Stella et al., 2012 [12]	France, 1992–2010	12	29 (19–38)	Mixed NSGCT: 75%. Teratoma component: NR Chorio: 16.7%. EC: 8.3%	NR	BEP	MT: 91.7% MT + LMS: 8.3%	NR	225 (10-4000)	100% -Aorta sectioning and anastomosis: 6 -IVC resection: 3 -Aorta sectioning and anastomosis + IVC resection: 1 -Infrarenal aortic graft + nephrectomy: 2	25% CD ≥ III: 25% -Small bowel occlusion and surgery: 1 -Perforated duodenal ulcer and surgery: 1 -Lymphocele requiring drainage: 1	15 (9–50)	59 (10–262)	Disease-free: 83.3%. Recurrence: 8.3% Death: 8.3% (melanoma)
Spiess et al., 2007 [11]	USA, 1980–2003	9	19 (16–53)	Mixed NSGCT: 89% Teratoma component: NR MT: 11%	5 (3–21)	NR	MT: 100%	432 (276–990)	600 (200-11650)	22% -Aortic repair: 1 -Ureteral repair: 1	44% CD ≥ III: 22% -Chylous ascites with drainage: 1. -Death due to sepsis: 1	NR	24 (7-137)	Disease-free: 77.8% 5-year OS: 89%
Andre et al., 2000 [10]	France, 1985–1997	30	29 (15–38)	MT: 86% Mixed NSGCT: 10.7%. Teratoma component: NR Seminoma 3.3%	20 (5–66)	BEP	MT: 100%	NR	NR	10% -IVC thrombectomy: 3 -Nephrectomy: 1	NR	NR	48 (3–80)	Alive: 90% Disease-free: 66.6%
Maroto et al., 1997 [9]	Spain, 1979–1994	11	21 (16–50)	Mixed NSGCT: 90.0%. Teratoma component: 82% EC: 9.1%	9 (7–13)	BEP, PVB, BOMP-EPI	MT: 100%	NR	NR	NR	NR	NR	103 (33–179)	Alive: 100% Disease-free: 54.5%
Ravi et al., 1995 [8]	India, NR	3	38 (32–48)	Mixed NSGCT: 100% Teratoma component: 100%	NR	BEP, IPVB	MT: 100%	NR	NR	NR	NR	NR	22 (6–26)	All alive and disease-free
Tongaonkar et al., 1994 [7]	India, NR	4	24 (15–33)	Mixed NSGCT: 100% Teratoma component: 75%	NR	BEP, VAB-6	MT: 100%	NR	NR	NR	NR	NR	21 (6–36)	All alive and disease-free
Jeffery et al., 1991 [2]	United Kingdom,1977–1990	13	25 (17–47)	Mixed NSGCT: 82%. Teratoma component: 77% Unknown: 8% (chemotherapy)	2 (1–25)	BEP, PVB, POMB/ACE,	Teratoma: 100%. -AC: 7.7%	NR	NR	NR	NR	NR	20 (1–68)	Disease-free: 80%. Alive: 92.3%
Tonkin et al., 1989 [6]	UK, 1977–1987	8	21 (15–26)	NSGCT: 100% Teratoma component: NR.	4 (1–6)	POMB/ACE	MT: 100%	NR	NR	NR	12.5% CD ≥ III: 12.5% -Death from aortic rupture:1	NR	34 (8–60)	Alive and disease-free: 75%
Logothetis et al., 1982 [1]	USA, 1971–1980	6	22 (16–32)	Mixed NSGCT 83.3%. Teratoma component: 83.3% EC − 16.7%	15 (8–19)	#	NR	NR	NR	NR	NR	NR	20 (5-108)	All alive and disease-free

AC: adenocarcinoma; B-COMF: Bleomycin, Cyclophosphamide, Vincristine (Oncovin), Methotrexate, 5-Fluorouracil; BEP: Bleomycin, Etoposide, Cisplatin; CD: Clavien-Dindo classification; Chorio: choriocarcinoma; CISCA: Cyclophosphamide, Adriamycin (doxorubicin), Cisplatin; EC: Embryonal carcinoma; EBL: Estimated blood loss; EP: Etoposide, Cisplatin; FU: Follow-up; IPVB: Ifosfamide, Cisplatin, Vinblastine, Bleomycin; LOS: Length of stay; LMS: leiomyosarcoma; max: maximum; min: minimum; MT: Mature teratoma; NSGCT: Non-seminomatous germ cell tumor; OS: Overall survival; P: Cisplatin; PEI: Cisplatin, Etoposide, Ifosfamide; POMB/ACE: Cisplatin, Vincristine, Methotrexate, Bleomycin / Actinomycin D, Cyclophosphamide, Etoposide; PVB: Cisplatin, Vinblastine, Bleomycin; RPLND: Retroperitoneal lymph node dissection; TIP: Paclitaxel, Ifosfamide, Cisplatin; VB: Vinblastine; VAB-6: Vinblastine, Actinomycin D, Bleomycin, Cisplatin, Cyclophosphamide, Hydroxyurea; VIP: Etoposide, Ifosfamide, Cisplatin; XRT: Radiation therapy; YST: Yolk sac tumor; # VB + P; XRT, VB + P, B-COMF; VB, Adriamycin, Bleomycin, VP-16; VB, B-COMF; VB + P, B-COMF; Adriamycin, Bleomycin, VP-, CISCA II, VB

### Evidence synthesis

The incidence of GTS among patients with NSGCT treated with chemotherapy was reported to range from 2.8% to 7.6% (2 studies) and the median age of presentation ranged from 16 to 38 years. Mixed NSGCTs were observed in 73–100% of cases (15 studies), with a teratoma component identified in 40–100% of these mixed tumours (7 studies). The teratoma component within mixed NSGCTs was 50–80% of the tumour volume (1 study) and pure mature teratoma was observed in 9–11% of orchidectomy specimens (3 studies). The median interval until RPLND ranged from 2 to 30 months. Most studies reported the use of BEP chemotherapy prior to RPLND (11 studies), with chemotherapy interruption required in 4.5% due to compressive symptoms (1 studies). Symptoms associated with the enlarging mass were reported in 50% to 83.3% of cases, with documented growth rates ranging from 0.5 to 0.7 cm per month (2 studies).

Pathological evaluation of RPLND specimens revealed mature teratoma in 91.7–100% (14 studies), with somatic-type malignant transformation observed in 7.7–8.3% of cases (2 studies). The median operative time for RPLND ranged from 160 to 432 min (4 studies), and estimated blood loss (EBL) varied from 225 mL to 2500 mL (5 studies). Adjunctive procedures were required in 10% to 100% of patients and median length of hospital stay (LOS) ranged from 5 to 15 days. (4 studies). Postoperative complication rates ranged from 12.5% to 44%, with Clavien-Dindo grade ≥ III complications occurring in 12.5% to 25% of patients (5 studies).

Follow-up ranged from 8 to 103 months (14 studies). At last follow-up, disease-free survival ranged from 41.7% to 100%, and overall survival from 73.7% to 100% (15 studies).

## Discussion

GTS remains a rare but clinically significant scenario in patients with NSGCT, with its pathogenesis still not fully understood. Despite the time elapsed since its initial description in 1982, limited published data continues to hinder consensus on its management and biological behaviour. Several hypotheses have been proposed to explain the development of GTS. One suggests that chemotherapy alters the kinetics of totipotent malignant germ cells, prompting their transformation into benign mature teratomatous components [19]. The retroconversion theory posits that chemotherapy destroys only the immature malignant cells, leaving behind mature benign teratomatous elements that continue to grow [20]. A third hypothesis considers the possibility of spontaneous differentiation of malignant cells into non-malignant components during treatment [21]. More recently, a molecular model has been proposed in which embryonal carcinoma cells differentiate into teratoma-forming transit amplifying cells capable of self-renewal or further differentiation into tissues of all three germ layers [4]. A recent study integrating transcriptional and proteomic profiling with tumour growth rate stratification proposed that specific biomarkers may aid in identifying GTS and anticipating its growth behaviour. Also, the authors advocate for an updated definition of GTS, describing it as a “continuously growing teratoma that might harbour occult non-seminomatous components considerably reduced during therapy but outgrowing over time again” [22]. Together, these theories highlight the complex and multifactorial nature of GTS pathogenesis.

Our systematic review aggregates data from 15 studies over five decades, involving 156 patients with an incidence up to 7.6% of patients with NSGCT treated with chemotherapy [2, 6]. Teratoma was identified in most orchidectomy specimens, although it was not always the predominant histological component, and some authors have questioned its predictive value for subsequent GTS development [4]. Teratoma was consistently present in the RPLND specimens, with two studies reporting cases of somatic-type malignancy, which is a recognised risk associated with teratoma progression.

Due to the resistance of GTS to both chemotherapy and radiotherapy, RPLND remains the cornerstone of treatment and is generally performed after systemic therapy is completed. However, over half of patients may experience compressive symptoms such as abdominal pain, acute renal failure, obstructive pyelonephritis or bowel obstruction [12, 15]. In rare cases, chemotherapy may need to be interrupted to allow surgical intervention due to life-threatening organ compression, as reported in one study (4.5%) [14]. Radiologically, these masses appear partially cystic, often multiloculated, with enhancing septations, progressive enlargement, and expanding cystic areas [17, 23]. The growth pattern is highly unpredictable, though some reports document rates between 0.5 and 0.7 cm per month [11, 13].

The surgical complexity is significant, with studies reporting high rates of adjunctive procedures including nephrectomies, bowel and major vascular resections, which reflects the local behaviour of GTS despite its benign histology. However, the wide range of adjunctive procedure rates (10% to 100%) likely reflects differences in patient selection, as the study reporting 100% involved a vascular surgery-led cohort that may not be representative. When excluding this outlier, reported rates range from 10% to 33%. The broad variation in median operative time (160–432 min), blood loss (225–2500 mL), and length of hospital stay (5–15 days), along with major postoperative complications (Clavien-Dindo ≥ III) reported in up to 25% of patients, further underscore the procedural heterogeneity and technical challenges involved. The wide interval to RPLND (8–103 months) may reflect a spectrum of GTS phenotypes, ranging from early aggressive to late indolent presentations. However, none of the included studies evaluated whether timing influenced surgical complexity or clinical outcomes.

From an oncological perspective, disease-free and overall survival rates were high. However, details regarding management of recurrence after RPLND were underreported, particularly the need for additional surgical resection or systemic therapy.

This systematic review has several limitations. All included studies were retrospective case series with limited patient numbers and inconsistent follow-up durations. There was substantial heterogeneity in the reporting of surgical outcomes, histopathological details, and follow-up, resulting in uneven data availability with some outcomes being reported in only one study, while others were consistently documented across all included studies, which also precluded a meta-analysis. Moreover, the risk of bias across the included studies imposes caution when interpreting the findings. The rarity of the condition, combined with inconsistent reporting, underscores the challenges in drawing conclusions. Data about reporting extra-abdominal manifestations of GTS were excluded, although it is noteworthy that the syndrome can present in diverse locations including the lungs, cervical and inguinal lymph nodes, mediastinum, forearm, mesentery, liver, and pineal gland [18, 23, 24]. These presentations often require distinct management strategies beyond the urological domain, although multidisciplinary surgical collaboration can be necessary to achieve complete resection.

This syndrome rarity and complexity further highlight the importance of centralizing testicular cancer care in high-volume, specialized centres with experienced multidisciplinary teams [25]. Multicentre collaborative databases and prospective registries are crucial for generating high-quality, standardised data on patients with GTS in the context of testicular cancer.

## Supplementary Information

Below is the link to the electronic supplementary material.

Supplementary Material 1

Supplementary Material 2

Supplementary Material 3

## Data Availability

All studies and respective data are included in the article and its supplementary material .
