# Supplementary material for: Retroperitoneal lymph node dissection for growing teratoma syndrome in testicular cancer: a systematic review of surgical outcomes
Source: World J Urol. 2026 Jan 13;44(1):88. doi: 10.1007/s00345-026-06207-5 (PMC12799728; doi:10.1007/s00345-026-06207-5)
Supplement: Supplementary file 1 — Supplementary Material 1 [file 345_2026_6207_MOESM1_ESM.docx]

**Supplementary material 2 - Inclusion and exclusion criteria by PICOS framework**

- Population (P): Patients with testicular non-seminomatous germ cell tumours (NSGCT) diagnosed with retroperitoneal GTS.
- Intervention (I): Surgical resection via retroperitoneal lymph node dissection (RPLND) for GTS.
- Comparator (C): Not applicable.
- Outcome (O): study characteristics (year range, country, study design), patient and tumour characteristics (age, histology and teratoma rates at orchiectomy and RPLND, timing to RPLND and chemotherapy regimens), surgical metrics (operative time, blood loss, adjunctive procedures, complications by Clavien Dindo classification, length of hospital stay), and oncological outcomes (recurrence and disease-free survival).
- Study design (S): Case series, retrospective cohort studies, and prospective cohorts.
