# Supplementary material for: Retroperitoneal lymph node dissection for growing teratoma syndrome in testicular cancer: a systematic review of surgical outcomes
Source: World J Urol. 2026 Jan 13;44(1):88. doi: 10.1007/s00345-026-06207-5 (PMC12799728; doi:10.1007/s00345-026-06207-5)
Supplement: Supplementary file 2 — Supplementary Material 2 [file 345_2026_6207_MOESM2_ESM.docx]

**Supplementary material 3** - Risk of bias assessment for each included study.

| Study | D1 | D2 | D3 | D4 | D5 | D6 | D7 | Overall |
| --- | --- | --- | --- | --- | --- | --- | --- | --- |
| Prineethie et al., 2022^17^ | 🔴 | 🟠 | 🟢 | 🟢 | 🟡 | 🟡 | 🟠 | 🔴 |
| Hsieh et al., 2022^18^ | 🟠 | 🟠 | 🟡 | 🟡 | 🟡 | 🟢 | 🟠 | 🟠 |
| Acikgoz et al., 2021^16^ | 🔴 | 🟠 | 🟡 | 🟡 | 🟡 | 🟡 | 🟠 | 🔴 |
| Paffenholz et al., 2018^14^ | 🔴 | 🟠 | 🟡 | 🟡 | 🟡 | 🟡 | 🟠 | 🔴 |
| Scavuzzo et al., 2014^15^ | 🔴 | 🔴 | 🟢 | 🟢 | 🟡 | 🟢 | 🟠 | 🔴 |
| Lee et al., 2014^13^ | 🔴 | 🟠 | 🟢 | 🟢 | 🟡 | 🟢 | 🟠 | 🔴 |
| Stella et al., 2012^12^ | 🔴 | 🔴 | 🟡 | 🟡 | 🟡 | 🟢 | 🟠 | 🔴 |
| Spiess et al., 2007^11^ | 🔴 | 🟠 | 🟢 | 🟢 | 🟡 | 🟢 | 🟠 | 🔴 |
| Andre et al., 2000^10^ | 🔴 | 🟠 | 🟡 | 🟡 | 🟡 | 🟡 | 🟠 | 🔴 |
| Maroto et al., 1997^9^ | 🔴 | 🟠 | 🟢 | 🟢 | 🟡 | 🟢 | 🟠 | 🔴 |
| Ravi et al., 1995^8^ | 🔴 | 🔴 | 🟢 | 🟢 | 🟡 | 🟢 | 🟠 | 🔴 |
| Tongaonkar et al., 1994^7^ | 🔴 | 🟠 | 🟢 | 🟢 | 🟡 | 🟢 | 🟠 | 🔴 |
| Jeffery et al., 1991^2^ | 🔴 | 🟠 | 🟢 | 🟢 | 🟡 | 🟢 | 🟠 | 🔴 |
| Tonkin et al., 1989^6^ | 🔴 | 🔴 | 🟡 | 🟢 | 🟡 | 🟢 | 🟠 | 🔴 |
| Logothetis et al., 1982^1^ | 🔴 | 🟠 | 🟢 | 🟢 | 🟡 | 🟢 | 🟠 | 🔴 |

D1 = confounding; D2 = classification of interventions; D3 = selection into the study; D4 = deviations from intended interventions; D5 = missing data; D6 = measurement of outcomes; and D7 = selection of the reported result.
