# Supplementary material for: Retroperitoneal lymph node dissection for growing teratoma syndrome in testicular cancer: a systematic review of surgical outcomes
Source: World J Urol. 2026 Jan 13;44(1):88. doi: 10.1007/s00345-026-06207-5 (PMC12799728; doi:10.1007/s00345-026-06207-5)
Supplement: Supplementary file 3 — Supplementary Material 3 [file 345_2026_6207_MOESM3_ESM.docx]

**Supplementary material 1** – Search strategy

Pubmed/MEDLINE

"Growing Teratoma Syndrome"[tiab] OR "growing teratoma"[tiab] OR (teratoma*[tiab] AND (growing[tiab] OR enlarg*[tiab] OR increas*[tiab])) OR "mature teratoma*"[tiab])

AND

("Testicular Neoplasms"[Mesh] OR "testicular cancer"[tiab] OR "testicular tumo*"[tiab] OR testicular[tiab] OR testis[tiab] OR testes[tiab] OR "germ cell tumo*"[tiab] OR NSGCT[tiab] OR nonseminomatous[tiab])

Embase

1. 'growing teratoma syndrome':ti,ab OR 'growing teratoma':ti,ab OR (teratoma:ti,ab AND (grow* OR enlarg* OR increas*):ti,ab) OR 'mature teratoma':ti,ab

2. exp testis tumor/ OR 'testicular cancer':ti,ab OR 'testicular tumo*':ti,ab OR testis:ti,ab OR testicle*:ti,ab OR 'germ cell tumo*':ti,ab OR NSGCT:ti,ab OR nonseminomatous:ti,ab

3. 1 AND 2
